# Non-Solvent Induced Phase Separation (NIPS) for Fabricating High Filtration Efficiency (FE) Polymeric Membranes for Face Mask and Air Filtration Applications

**DOI:** 10.3390/membranes12070637

**Published:** 2022-06-21

**Authors:** Ebuka A. Ogbuoji, Lauren Stephens, Amber Haycraft, Eric Wooldridge, Isabel C. Escobar

**Affiliations:** 1 Department of Chemical and Materials Engineering, University of Kentucky, Lexington, KY 40506, USA; ebuka.ogbuoji@uky.edu (E.A.O.); lauren.stephens1@uky.edu (L.S.); amber.haycraft@uky.edu (A.H.); 2 Digital Printing Technology, Somerset Community College, Somerset, KY 42501, USA; eric.wooldridge@kctcs.edu

**Keywords:** filtration efficiency, aerosols, 3D printing, COVID-19, air filtration

## Abstract

Protection against airborne viruses has become very relevant since the outbreak of SARS-CoV-2. Nonwoven face masks along with heating, ventilation, and air conditioning (HVAC) filters have been used extensively to reduce infection rates; however, some of these filter materials provide inadequate protection due to insufficient initial filtration efficiency (FE) and FE decrease with time. Flat sheet porous membranes, which have been used extensively to filter waterborne microbes and particulate matter due to their high FE have the potential to filter air pollutants without compromising its FE over time. Therefore, in this study, single layer polysulfone (PSf) membranes were fabricated via non-solvent induced phase separation (NIPS) and were tested for airflow rate, pressure drop and FE. Polyethylene glycol (PEG) and glycerol were employed as pore-forming agents, and the effect of the primary polymer and pore-forming additive molecular weights (MW) on airflow rate and pressure drop were studied at different concentrations. The thermodynamic stability of dope solutions with different MWs of PSf and PEG in N-methylpyrrolidone (NMP) at different concentrations was determined using cloud-point measurements to construct a ternary phase diagram. Surface composition of the fabricated membranes was characterized using contact angle and X-ray photoelectron spectroscopy (XPS), while membrane morphology was characterized by SEM, and tensile strength experiments were performed to analyze the membrane mechanical strength (MS). It was observed that an increase in PSf and PEG molecular weight and concentration increased airflow and decreased pressure drop. PSf60:PEG20:NMP (15:15:70)% *w*/*w* showed the highest air flow rate and lowest pressure drop, but at the expense of the mechanical strength, which was improved significantly by attaching the membrane to a 3D-printed polypropylene support. Lastly, the FE values of the membranes were similar to those of double-layer N95 filters and significantly higher than those of single layer of N95, surgical mask and HVAC (MERV 11) filters.

## 1. Introduction

Air filters are vital for improving air quality by trapping dust, particulate matter, allergens, and other materials that may be present in the atmosphere. Applications of air filters can be found in various industries, such as medical, commercial, automobile and military [1]. The importance of high efficiency particulate air filters (HEPA) for HVAC systems and face masks have been emphasized in recent times due to the SARS-CoV-2 pandemic for effective protection against aerosolized microbes. The use of these high filtration efficiency (FE) filters has reportedly resulted in a drastic decrease in infection rate of the easily transmissible virus (SARS-CoV-2) [2,3]. High efficiency particulate air filters (HEPA), which can trap 99.95% of dust particulates and aerosolized microbes greater than 0.3 µm in the air, have been recommended by the American Institute of Architects (AIA), the Centers for Disease Control (CDC) and the American Society of Heating Refrigerating and Air Conditioning Engineers (ASHRAE) for optimal protection against aerosolized microorganisms [4]. Similarly, high FE face masks such as N95 have been recommended by the CDC for maximum protection against aerosolized pathogens [5]. Air filters for HVAC systems are typically made from fiber glass, polymer fiber and pleated cotton while face mask filters are made using nonwoven fibers, which have web-like structures formed by entangled fibers from various synthetic polymers such as polypropylene, polyethylene, polystyrene, polyurethane and polyacetonitrile [6,7,8]. These nonwoven fibers used for face mask production are usually produced via spun bonding, melt blowing, and electrospinning, and have important characteristics, such as low air resistance, versality and multiple layers [9,10].

Two major mechanisms governing air filtration are mechanical/physical and electrostatic mechanism [7]. Physical or mechanical mechanisms include diffusion, interception, inertia impaction, straining/sieving and gravity sedimentation [11]. These mechanisms affect the filter FE and are a function of filtration velocity and particle size [12]. Fiber glass, which has been used extensively as HVAC filters and non-woven fibers, achieve high filtration efficiency by electrostatic interaction [7,13]. This filtration mechanism allows for high initial filtration efficiency with low pressure drop. Commercially available face masks such as N95 and KN95 achieve high breathability without compromising the FE by electrostatic attraction [12,14,15]. The downside of this mechanism is that the charged media is often short-lived since various environmental factors such as humidity, corrosive vapors, and salt can cause a premature discharge or charge masking, resulting in reduced filtration efficiency performance [16]. This makes mechanical mechanism more attractive for critical filtration applications such as protection against pathogenic microbes.

Polymeric membranes have received increased attention in the past decade for high FE applications using mechanical filtration because the tortuosity and asymmetry of membrane pores can help increase FE at low-pressure drops [17]. In addition, controllable thickness, pore size, porosity and tunable surface functionalization make polymeric membranes very attractive for air filtration [17]. HEPA filters capture aerosolized microbes efficiently; however, these filters can become breeding grounds for microbes due to the suitable conditions, i.e., temperature and humidity present in the filter environment [4]. These microbes tend to multiply effectively in the filters by using other trapped particulates as food sources [4]. The resulting offspring are then dispersed back into the air, causing health and safety concerns [4,18,19,20]. Cleaning these filters can result in FE reduction, and inappropriate disposal can lead to environmental problems. The modifiable surface and pore composition of polymeric membranes enhance functionalization with antimicrobial agents, such as silver, oxides of copper, zinc, and titanium [21,22,23], making membranes a potential candidate for manufacturing reusable filters for HVAC systems and face mask applications.

**Figure 1:**
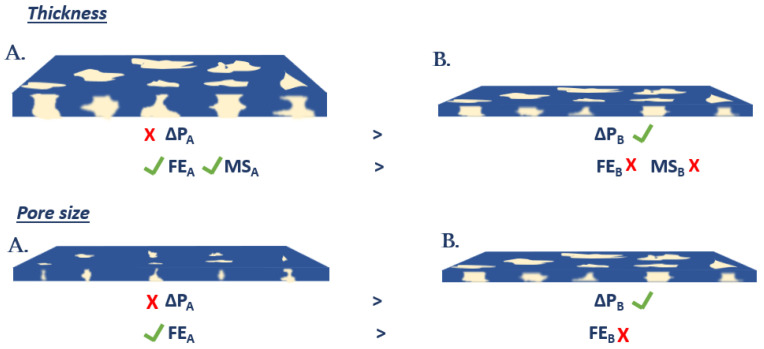
Schematic showing factors affecting pressure drop, filtration efficiency and mechanical strength.

In fabricating membranes for air filtration, factors such as FE and pressure drop must be considered [24]. These parameters are dependent on membrane thickness and pore sizes, as shown in Figure 1. Reducing the membrane thickness results in reduced airflow resistance and pressure drop but can compromise the mechanical strength (MS). This relationship follows Darcy’s law (Equation (1)):

(1) $$\frac{Q}{A}=-\frac{x}{\eta }.\frac{∆P}{e}$$

where *Q* is the volumetric flow rate, *A* is the normal cross-sectional area, ∆*P* is the pressure drop, *e* is the membrane thickness, *x* is the intrinsic air permeability, and *η* is the viscosity.

Pore size can also affect the filtration efficiency and pressure drop across the membranes since pore size determines the size of particles filtered and resistance to airflow (Figure 1). Another factor affecting pressure drop is the membrane thickness, and to attain a very low pressure drop, the membrane thickness might range between 50 and 250 µm, which may not withstand the mechanical stress from heavy breathing and mask mishandling. Therefore, optimal thickness and pore size must be achieved to ensure sufficient FE, minimal pressure drop and good mechanical strength.

To fabricate face masks/air filters using membranes with minimum thicknesses, porous mesh supports have been proposed to provide external mechanical strength (MS). MS of membranes has been reportedly improved using nonwoven supports made from polyester fibers [25,26]. Additive manufacturing or 3D printing can provide an alternative for fabricating membrane macroporous supports. 3D printing involves using materials, such as polymers and metals to create a three-dimensional object [27]. Some advantages of 3D over traditional manufacturing processes include reduced product development cost, small scale production cost-effectiveness, rapid manufacturing of finished components, improved product quality, prevention of downtimes during production and manufacture of complex geometries [28]. The process of 3D printing an object starts by designing the object model using computer-aided design (CAD) software, which is then converted into a standard triangle language (STL) file format responsible for storing information on the 3D object surface as coordinates of triangular sections [29]. The 3D model is then spliced into several 2D cross-section layers and sent to the 3D printer to process [29]. Fused deposition modeling (FDM) is the most common method used for 3D printing. This involves the continuous melting and extrusion of a thermoplastic filament before layer by layer deposition on a growing work [29].

This study explored the use of porous flat sheet PSf membranes made via NIPS for air filtration applications such as face mask and HVAC filters. Aerosolized viruses, such as SARS-CoV-2 aerosols, have been found between two size ranges (0.25–1 µm) and >2.5 µm [30], which makes microfiltration and ultrafiltration membranes perfect for their capture. Therefore, this work aimed to fabricate membranes that match or exceed filtration efficiencies of commonly used air filters for sustained protection against aerosolized microbes by exploiting polymer-pore former conformation and thermodynamic stability of casting solutions to ensure increased porosity and air flow rate. In this work, the effect of the primary polymer and pore former molecular weights at various pore former concentrations on casting solution thermodynamic stability, air permeability, FE and ∆P were investigated. Different molecular weights (MW) of polyethylene glycol (PEG) were used as pore-former additives. These hydrophilic additives interact with water molecules in hydrophobic PSf casting solution during NIPS, resulting in the nucleation of emulsion drops which forms stable pores [31,32]. The effect of different PEG MWs and concentrations on air filtration and membrane mechanical strength were also analyzed. Lastly, the use of a 3D printed macroporous supports to provide the membrane-based air filters with mechanical strength was also investigated. Although PSf and PEG have been used extensively to fabricate membranes via NIPS for water purification and gas separation, these materials and technique have not been significantly explored for air filtration. Furthermore, this work studied the use of a 3D printed support for air filter membrane fabrication.

## 2. Materials and Methods

Polysulfone (PSf) pellets with average molecular weights of MW 35,000 Da (PSf35) and MW 60,000 Da (PSf60) were obtained from Sigma Aldrich (Saint Louis, MO, USA) and Acros Organics (Carlsbad, CA, USA), respectively. PEG with MW of 1000, 4000, 8000, 10,000 and 20,000 Da were purchased from Alfa Aesar (Haverhill, MA, USA). N-Methylpyrrolidone (NMP) with 99% purity was purchased from VWR Chemicals (Radnor, PA, USA).

### 2.1. Dope Solution Preparation and Casting Method

The primary polymer (PSf) and pore-forming additives were dissolved in NMP at concentrations shown in Table 1. Solutions with additives (MW ≤ 10 kDa) were stirred at room temperature while those with additives (MW > 10 kDa) were heated to 50 °C and stirred at 100 rpm in a tightly sealed container until a clear solution was obtained, signifying complete dissolution. The dissolved solution was sonicated to remove bubbles and kept at room temperature for 30 min before casting using an automatic bench-top flat sheet casting machine (Model: BTFS-TC, PMI, Ithaca, NY USA) set at a casting speed of 500 cm/min to avoid inconsistencies in membrane thickness which could result from casting manually [33]. The casted solution was then immersed in a coagulation (water) bath at room temperature for 15 min before transferring into a container with deionized (DI) water, where it was stored for 24 h. The membrane was then dried in a convective dryer set at 30 °C for 24 h and was tested afterwards.

### 2.2. Airflow, Pressure Drop and Filtration Efficiency (FE) Test

**Figure 2:**
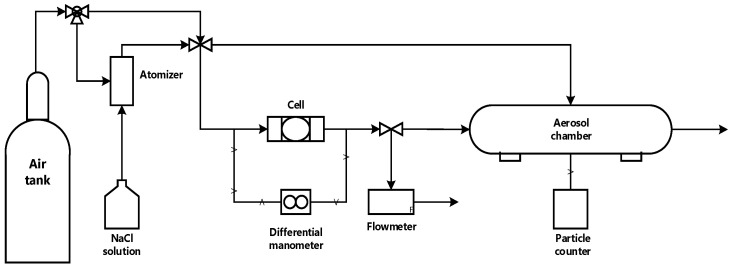
Schematic for airflow, pressure drop, and filtration efficiency test setup.

The completely dried samples were cut into spherical shapes (4.5 cm diameter) to fit a 47 mm in-line polycarbonate filter holder (Pall corporation, Show Low, AZ, USA) as shown in Figure 2. The cell was connected to an air source (high purity compressed air), a differential manometer (VWR, Radnor, PA, USA) was connected across the cell to measure pressure drop, and a digital mass flow meter purchased from Kelly Pneumatics (Newport Beach, CA, USA) was connected to the end of the system. Air resistance and pressure drop tests were conducted at 0.4 and 0.55 bar since these pressures result in flowrates greater than reported normal human respiration flowrate (6–10 LPM) calculated by multiplying normal breathing tidal volume (0.5 L [34]) by normal breathing rate (12–20 breaths/minute [35]). Experiments were performed in triplicates to ensure data reproducibility.

The FE test was conducted in the setup depicted in Figure 2. In this setup, a 100 ppm aqueous sodium chloride (NaCl) solution was connected to a constant output atomizer (model 3076, TSI incorporated, MN, USA), which generated a distribution of aerosol particle sizes [17]. Pressure from an air source acted as the driving force for drying and transporting the generated aerosols across the system. A filter holder (cell) was placed between the airflow stream and an ~3 in internal diameter cylindrical PTFE tube with a particle counter (Met One model GT-526S, Grant Pass, OR, USA) operating in differential mode to estimate counts of permeate particles sizes through the filter/membrane in the cell. The test was conducted at 30 ± 3 liters per minute (LPM), flow rates greater than normal breathing conditions [34,35]. Particle counts were collected after the system was allowed to equilibrate for 1–3 min. The PTFE tube was air-flushed for 1–2 min in between experiments to remove unsettled NaCl aerosols. Filtration efficiency was estimated by Equation (2).

(2) $$FE=\frac{E_{m}-F_{m}}{E_{m}}\times 100$$

where $E_{m}$ and $F_{m}$ are particle counts through empty cell and occupied cell, respectively.

### 2.3. Tensile Test, Membrane Wettability and Cloud Point Measurements

Fabricated membrane mechanical strength was obtained using a 5 kN Instron tensile tester (model 4465, Norwood MA, USA). Samples were pulled under an applied force to obtain a tensile profile before material failure. Dog bone shapes (narrow middle and wider end) of the samples were clamped unto the tester (image in supplementary materials section Figure S5). The sample with a midsection dimension of (15 × 5) mm was stretched at a constant speed of 5 mm/min. The tensile load was applied incrementally until a fracture occurred at the midsection. The tensile force and the length change were recorded automatically as the experiment proceeded. All tests were performed in triplicate, and results were averaged.

The membrane wettability was determined by using a drop shape analyzer (Kruss DSA100, Matthews, NC, USA) by estimating the contact angle between a sessile drop and a flat membrane sample. Contact angle correlates with hydrophobicity such that as contact angle increases, hydrophobicity increases. Face masks with high hydrophobicity are preferred since water retention in pores can cause respiration difficulty [36]. To measure contact angle, 20 µL of water was pipetted on the membrane attached to a flat glass plate and was mounted firmly on a horizontal stage (supplementary materials Figure S3). Baseline (boundary line) adjustments between the drop contour and the membrane surface conducted on the instrument software were consistent for each experiment, measurements were taken after 10 s to ensure liquid equilibration on the solid surface, and a 20 µL was used to reduce the effect of impurities. The test was performed on three different regions of the sample, and the mean was reported.

To investigate the thermodynamic stability of the casting solutions, a phase diagram was constructed by measuring cloud point. Cloud point measurements were carried out by the titration method, which involves adding water into a solution until a visually turbid solution is formed [37]. Homogeneous polymer casting solutions were first obtained at 50 °C and 150 rpm before adding water. Ultrapure water was then dropped into the homogeneous solution at 70 °C and 150 rpm until a visually cloudy solution was obtained. On adding water, the solution was agitated using a vortex shaker when localized precipitation occurred until the precipitated chunk disappeared. Cloudy solutions were maintained at operating conditions (70 °C and 150 rpm) for 30 min to confirm turbidity. Once a turbid solution was confirmed (cloud point), the compositions of the solutions were recorded and used to construct a ternary phase diagram.

### 2.4. Porosity and Viscosity Measurements

Membrane porosity was estimated by the gravimetric method, which involves weighing dry and wet membrane samples to obtain a weighted average [38]. Fabricated membrane samples were cut into 1 × 1 cm^2^ sheets and were soaked in isopropanol (IPA) for 24 h. The soaked flat sheets were then weighed wet and dried in an oven at 50 °C for 24 h. Dried membranes were weighed after drying completely, and the membrane porosity (P) was calculated using Equation (2) described by [39]:

(3) $$P=\frac{V_{i}}{V_{t}}=\frac{\frac{\left(m_{w}-m_{d}\right)}{\rho_{i}}}{\frac{\left(m_{w}-m_{d}\right)}{\rho_{i}}+\frac{m_{d}}{\rho_{m}}}\;$$

where $V_{i}$, $V_{t}$, $m_{w}$, $m_{d}$ $\rho_{i}$ and $\rho_{m}$ are IPA volume, total volume, wet membrane weight, dry membrane weight, IPA density and membrane density, respectively. The experiment was performed in triplicate to ensure reproducibility.

Casting solution viscosity was obtained using a rheometer (AG-G2, TA Instruments, New Castle, DE, USA) in a parallel plate geometry (40.0 mm parallel plate, Peltier plate stainless steel). Viscosity measurements were taken at 25 °C and a shear rate of 0.1 1/s.

### 2.5. X-ray Photoelectron Spectroscopy (XPS), Scanning Electron Microscope (SEM), and Surface Pore Analysis

XPS was used to obtain a quantitative elemental composition of the fabricated membranes. XPS characterization was performed using a K-Alpha XPS apparatus equipped with an A1 K (1486.6 eV) source (Thermo Fisher Scientific, Waltham, MA, USA). The X-ray source had an accelerated voltage of 10 kV, an emission current of 12 mA, and the takeoff angle of the photoelectron was set at 90°. Peaks of carbon, oxygen, and sulfur were fitted using an Avantage software version 5.9918, Thermo Fisher Scientific, Waltham, MA, USA. A depth profile analysis was also performed with the same equipment to obtain the elemental composition of the membrane matrix below the surface layer. A 200 eV ion beam was used to etch 100 nm layer of the membrane top surface for 5 cycles at 60 s/cycle, and the elemental compositions of each sample were measured.

Fabricated membrane morphology was observed by SEM (FEI Helios Nanolab 660, Thermo Fisher Scientific, Waltham, MA, USA). The samples were prepared by first fracturing in liquid nitrogen and then sputter-coated with platinum before observations were carried out on the membrane cross-section.

SEM images of different magnifications (1000×, 2500×, 5000×) were used to analyze surface pore characteristics. Images were obtained using SEM (FEI Helios Nanolab 660, Thermo Fisher Scientific, Waltham, MA, USA) and were further analyzed using ImageJ, an open-source image analysis software to obtain average pore size and pore size distribution. Pore size was obtained manually after adjusting the image scale by measuring the pore diameter. Pore sizes were measured for three magnifications 1000× (50 µm), 2500× (10 µm), and 5000× (5 µm) for each membrane, and were averaged to obtain the mean pore size. Other surface images and pore size distributions were obtained and reported in the supplementary materials (Figures S1, S6 and S7).

## 3. Results and Discussion

### 3.1. Membrane Composition

To determine the elemental membrane surface composition, the membranes were characterized using X-ray photoelectron spectroscopy (XPS), as shown in Table 2. An increase in surface oxygen concentration was observed in the membranes fabricated using casting solutions containing PEG. PSF35:PEG20 (P7) and PSF60:PEG20 (P9) both showed higher oxygen contents of 15.55 ± 1.7 mol% and 15.75 ± 1.05 mol%, respectively, as compared to 12.74 ± 0.68 mol% for PSf35 (P1) and 12.23 ± 0.29 mol% for PSF60 (P2). Additionally, oxygen/carbon ratios were higher for P7 (0.188) and P9 (0.192) compared to their pristine counterparts P1 (0.148) and P2 (0.141). The increase in membrane surface oxygen is hypothesized to originate from the oxygen in PEG backbone and terminal hydroxyl functional group, which suggests that the additive does not completely diffuse into the non-solvent during phase separation, instead, some PEG remnants are trapped in the membrane matrix. Different mechanisms of pore formation in the presence of water-soluble pore-forming additives during non-solvent induced phase separation (NIPS) have been proposed in literature and are summarized here [31,40,41,42]. Some studies suggest that additives cause delayed solidification of membrane matrix resulting from pore former diffusion into the non-solvent (water), leading to the formation of fully developed macropores [41,43]. Other studies suggest water-soluble additive migration to the surface layer of the membrane [40,42], which could support the observation here of an increased amount of oxygen on the surface of the membrane potentially arising from the pore former.

**Figure 3:**
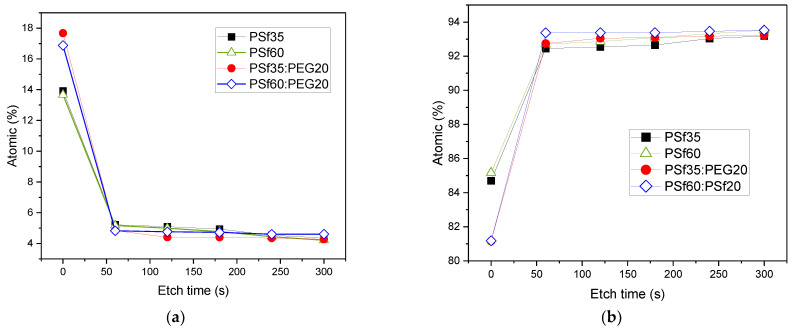
Depth profile of fabricated PSf membranes, (**a**) oxygen (O1s) profile, and (**b**) carbon (C1s) profile.

To further investigate the hypothesis of PEG entrapment in the membrane matrix, a depth profile was obtained using XPS by etching the first five 100 nm top layer of the membrane surface. For all samples, oxygen concentration decreased with depth; however, P7 and P9 had a higher decrease in oxygen content, 71.4% and 72.6% respectively, between the top surface and the first etched surface layer compared to P1 (62.5%) and P2 (62.2%) (Figure 3a and Table 3), which implies a higher surface oxygen concentration for P7 and P9. Beyond the first etch layer, there was no significant difference between the oxygen content of the etched layers for all samples (Figure 3a). This indicates that PEG, responsible for the increased oxygen content, is only present in the surface layer of the membrane, which agrees with the proposed reason that the pore former migrates to the surface during NIPS [40,42]. When the cast solution was immersed in water, the hydrophilic additive migrated to the membrane top layer in an attempt to diffuse into the non-solvent. However, instantaneous demixing occurred, leading to fast precipitation; hence, some long-chain PEG molecules were trapped and immobilized at the surface of the membrane. The presence of increased oxygen content on the surface layers of P7 and P9 resulted in decreased carbon concentrations on their surfaces as compared to P1 and P2. The percent increase in carbon concentration between the top surface and the first etched layer for P7 and P9 were higher compared to those of P1 and P2 (Figure 3b and Table 3); which again signifies a higher oxygen concentration at P7 and P9 membrane surfaces.

The higher oxygen composition on the membrane surface, observed in Figure 3a and Table 3, may condense humid air and wet the pores. Therefore, observing that PEG does not completely exit the membranes during NIPS is a key finding, and there are techniques that can be employed to address this, such as by surface grafting an organosilane to react with the hydroxyl group from the PEG on the membrane surface. For example, fluoroalkylsilane (FAS) has been used to increase membrane hydrophobicity by 300% [44]. The addition of surface modifying macromolecules (SMM) in dope solutions has also been reported to increase the hydrophobicity of polymeric membranes formed by phase inversion because the active SMM, which has a low polarity component, migrates to the top layer of the membrane during phase separation, thereby enhancing hydrophobicity without significantly compromising flux [45,46]. Other additives, such as clay nanocomposites, have reportedly increased hydrophobicity [47] if pore wetting becomes a problem.

### 3.2. Membrane Wettability

**Figure 4:**
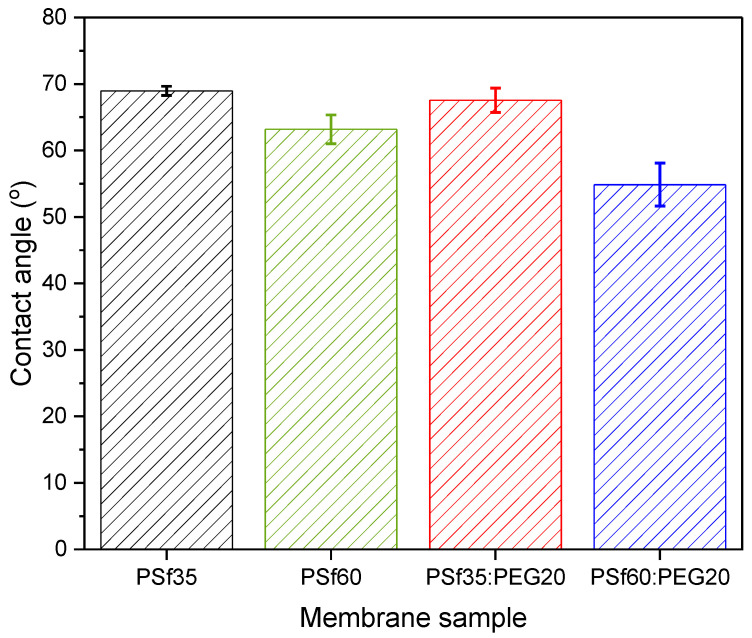
Contact angle measurements for fabricated PSf membranes.

Contact angle measurements were conducted to obtain the hydrophilicity or wettability of the membrane surface. Membranes fabricated with solutions containing pore forming additives showed a lower contact angle compared to their pristine polymer solution counterparts. As observed in Figure 4, the contact angle slightly decreased from 69 ± 0.69° for PSf35(P1) to 68 ± 1.8° for PSf35:PEG20(P7); however, the decrease is not statistically significant. On the other hand, for the higher molecular weight PSf, the contact angle decreased from 63.18 ± 2.2° for PSf60(P2) to 54.87 ± 3.2° for PSf60:PEG20(P9). Contact angle measurements agree with Table 2, which showed an average increase of approximately 3 mol% in the amount of oxygen between P1 and P2 with the addition of PEG pore former. Since PEG is a hydrophilic compound, the decrease in contact angle further strengthens the hypothesis that the pore former migrates to the surface of the membrane during phase inversion and is entrapped due to fast precipitation rate. Barambu et al. [42] and Zhu et al. [40] also reported a similar trend of decreasing contact angle in the membranes fabricated with solutions containing PEG. Furthermore, a decrease in contact angle with increasing PSf molecular weight as seen in Figure 4 for P7 (67.55 ± 1.8°) and P9 (54.87 ± 3.2°) could be due to slower PEG transport to the membrane surface and into the non-solvent caused by increased chain entanglement in the presence of longer polymer chains (i.e., PSf 60 kDa). This means there would be more PEG molecules at the P9 membrane surface than P7 since more PEG diffused into water from the latter, which agrees with the surface composition data in Table 2.

### 3.3. Effect of Pore Formers and Pore Former Molecular Weight on Air Permeability

**Figure 5:**
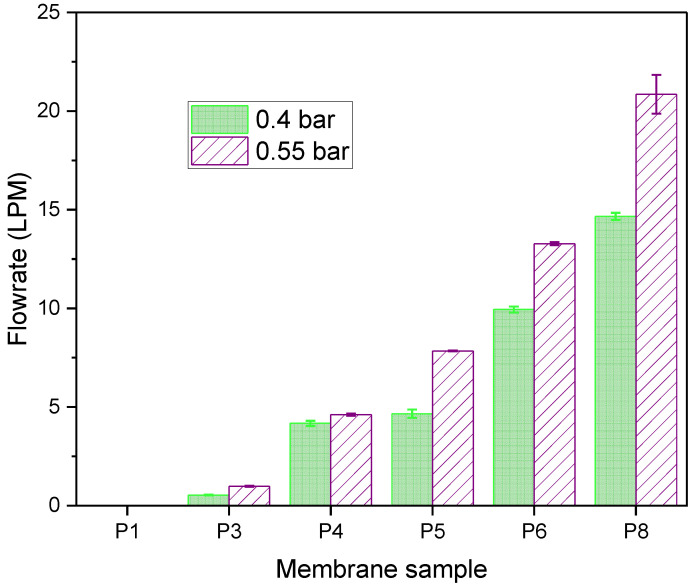
Air flow rate through fabricated PSf membranes.

Reduced airflow resistance across membranes can be obtained using additives, known as pore formers, which increase the pore size of the membrane top layer and the porosity of the membrane sublayer [31,32,48,49]. To study the effect of pore formers on air flow rate, porous flat sheet membranes were fabricated using casting solutions with and without additives. The PSf and pore former concentrations in each membrane were 15% (*w*/*w*) and 10% (*w*/*w*), respectively. For membranes made from purely PSf casting solution (P1), there was no air flow when subjected to high purity compressed air (Figure 5). Conversely, membranes made from casting solutions containing water soluble compounds or pore formers, such as polyethylene glycol (PEG, on P4–P6, P8) and glycerol (P3), showed significant air flow ranging from 0.5–20.9 LPM at 0.55 bar. The observed effect of additives on air flow is due to reduced thermodynamic stability of the casting solution which causes enhanced precipitation rate of membranes [31]. Faster precipitation during phase separation has been reported to result in membranes with larger pore sizes [31,50].

**Figure 6:**
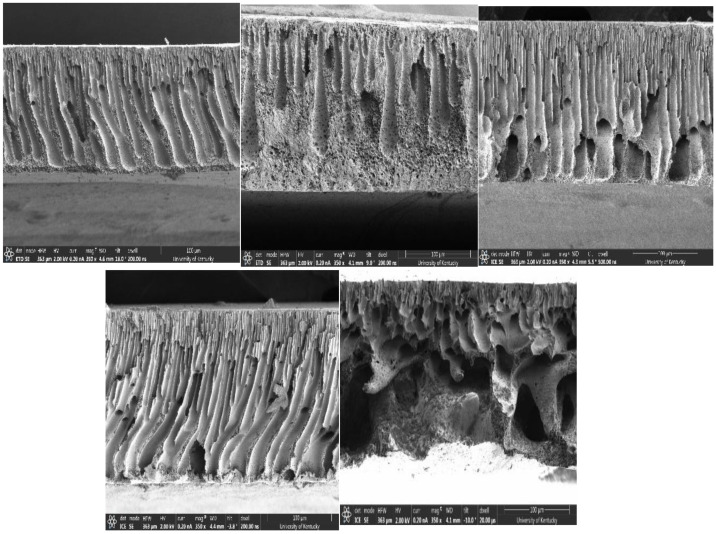
Cross sectional SEM images; Top left—P1, Top middle—P3, Top right—P4, Bottom left—P5, Bottom right—P8.

Furthermore, PEG, a flexible chain polymer, combines with PSf to reduce the miscibility of the casting solution with the non-solvent causing an instantaneous demixing during NIPS, and increased viscosity of the casting solution. Viscosity increases with the ratio of non-solvent inflow to solvent outflow, resulting in increased porosity of membranes [32,51]. The longer the chain length of PEG, the higher the probability of chain entanglement responsible for viscosity in higher molecular weight polymer solutions. As expected, P8 showed the highest air flowrate, which correlates with the increased porosity of the membrane (Figure 5). Membranes made from solutions with PEG additives showed increased flow rate with molecular weight from 4.2–14.7 LPM at 0.4 bar and 4.6–20.9 LPM at 0.55 bar. P3 showed lower flowrate compared to the other pore formers studied. This is because glycerol has a lower molecular weight compared to the PEGs studied, hence a lower viscosity leading to lower porosity and flowrate (effect of NaCl, another small molecular weight compound, was reported in the Supplementary Materials Table S1). Table 4 shows that the viscosity of the casting solution increased with increasing PEG molecular weight leading to varying sizes of fully/partially developed macrovoids in the membranesublayer, as also observed in Figure 6. Although all membranes showed finger-like pore structures resulting from instantaneous demixing, it is noticeable that there were more developed macrovoids in the presence of pore formers, which strengthens the effect of viscosity on macrovoid formation in the pore structure.

### 3.4. Effect of Pore Former Concentration on Airflow Rate and Pressure Drop

**Figure 7:**
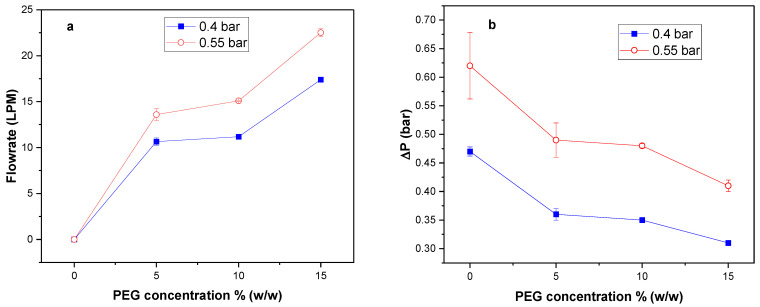
Effect of PEG concentration on (**a**) air flow rate and (**b**) pressure drop on PSf35:PEG20:NMP membranes.

To observe the effect of additive pore-former (PEG) concentration on membrane formation, varying concentrations of PEG were used in making porous flat sheet membranes. The concentration studied were 0, 5, 10, and 15% (*w*/*w*) PEG in membranes composed of 35 kDa PSf, 20 kDa PEG since porosity increases with additive MW [32], and NMP. Figure 7a shows that airflow through the resulting membranes increased with increasing PEG concentration, while pressure drop decreased with an increase in PEG concentration for all membranes (Figure 7b). P7, which had the highest PEG concentration (15% *w*/*w*), resulted in the highest flow rate and lowest pressure drop at both 0.4 bar (17.4 LPM, 0.31 bar) and 0.55 bar (22.5 LPM, 0.36 bar). The viscosity of PSf/PEG solutions has been reported to increase with PEG concentration [52], and as shown here in Table 4. The effect of additive concentration on airflow rate and pressure drop was likely caused by increased viscosity with increasing PEG concentration since viscosity has shown a directly proportional relationship with membrane porosity [51].

### 3.5. Effect of Polysulfone Molecular Weight on Airflow Rate and Pressure Drop

**Figure 8:**
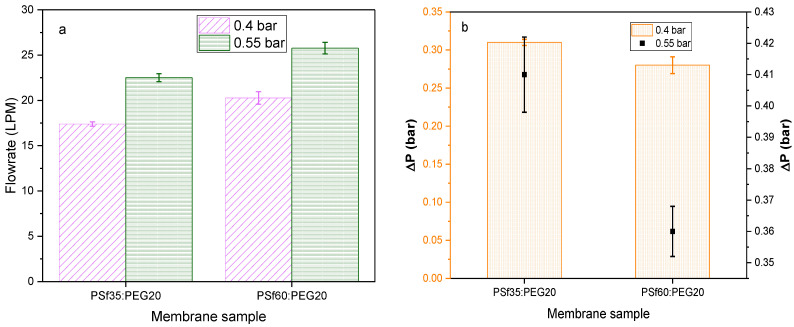
Effect of PSf molecular weight on (**a**) air flow rate; (**b**) pressure drop across PSf/PEG membranes.

**Figure 9:**
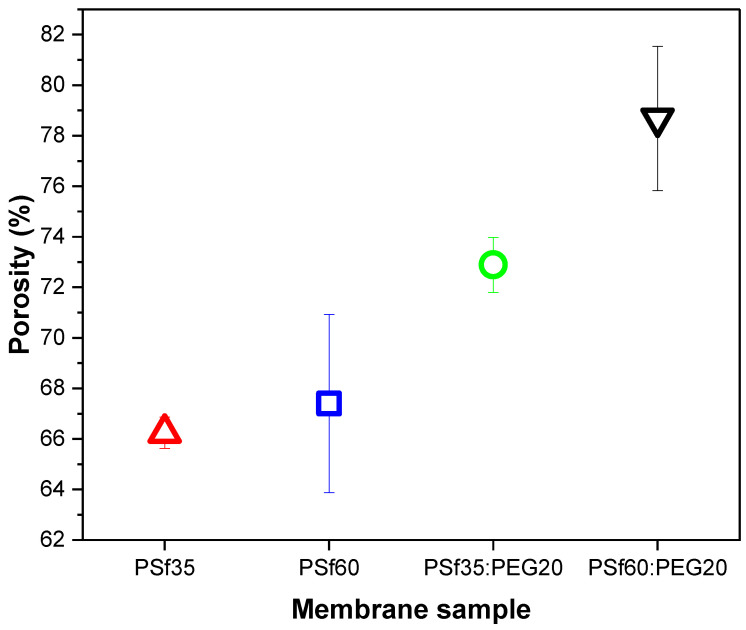
Porosity of fabricated membranes.

As mentioned previously, polymer molecular weight is estimated by the chain length in the polymer; thus, the higher the molecular weight, the longer the chains. Chain length can affect the arrangement of the polymer in a membrane matrix which can, in turn, affect porosity and flux across the membrane. To determine the effect of PSf molecular weight on air flow resistance and pressure drop, membranes of two PSf molecular weights (35 kDA and 60 kDA) were fabricated and tested. The membrane composition was kept at 15:15:70 (% *w*/*w*) of PSf:PEG20:NMP. Figure 8a,b shows that P9 had the highest airflow rate of 20.3 LPM at 0.4 bar and 25.8 LPM at 0.55 bar. The effect of PSf molecular weight on flow rate could be analyzed based on thermodynamics, kinetics, and polymer conformation. PSf60, a longer chain polymer compared to PSf35, would make a less compact conformation during precipitation in the presence of another long-chain polymer such as PEG20 because of the reduced potential to arrange properly. This could result in larger pores and increased porosity after NIPS since PEG diffuses into the non-solvent as precipitation occurs [42]. Figure 9 shows an increase in porosity with PEG molecular weight in fabricated membranes.

**Figure 10:**
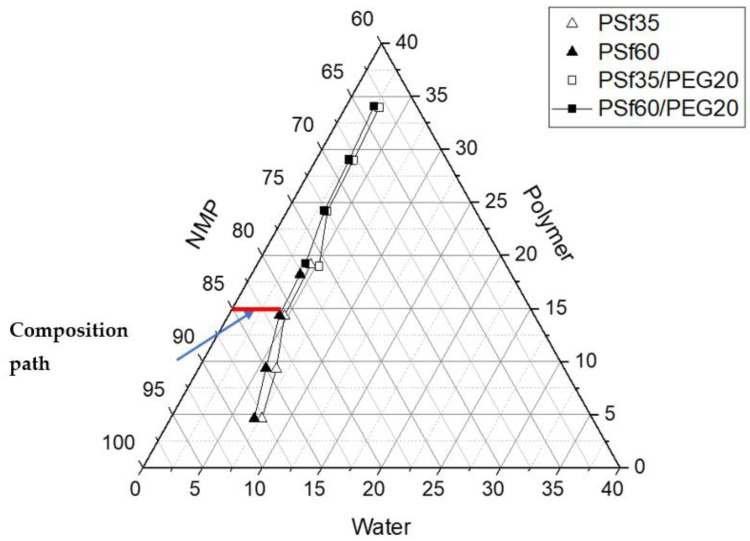
Casting solution ternary phase diagram.

In addition, viscosity increases with molecular weight leading to increased porosity and increased airflow rate. Viscosity showed an increasing trend with PSf molecular weight in casting solutions (Table 4) which resulted in more porous membranes, shown in Figure 9; i.e., P9 with average viscosity of 21.1 ± 4.21 Pa•s versus P7 (16.1 ± 3.54 Pa•s). Lastly, the thermodynamic stability of the casting solution reduced with increased PSf molecular weight (Figure 10) causing a rapid exchange between solvent and non-solvent, resulting in faster precipitation associated with more porous membranes [53]. Pressure drop is expected to correlate with airflow rate since both parameters relate to membrane resistance to airflow. For samples with lower resistance to airflow, there would be a higher flow rate and a lower pressure drop. This correlation can be seen in Figure 8b, where P9, which has the highest flow rate (20.3 LPM at 0.4 bar), showed the lowest pressure drop at both 0.4 bar (0.28 bar) and 0.55 bar (0.36 bar).

### 3.6. Ternary Phase Diagram

The decrease in thermodynamic stability with increasing molecular weight of both PSf and the presence of PEG can be seen in the ternary phase diagram (Figure 10). Thermodynamic stability has been reported to affect membrane pore structure, porosity, and mechanical strength of the membrane [31,37,54]. To investigate the thermodynamic stability of the casting solutions used in fabricating membranes in this work, a phase diagram was constructed by cloud point measurement. The phase diagram encompasses an experimental binodal curve (cloud point) which represents compositions of thermodynamic instability and phase transition. Cloud point curve for solutions without additives (PSf35 (P1) and PSf60 (P2)) and with additives (PSf35:PEG20 (P7) and PSf60:PEG20 (P9)) were obtained. For all solutions, the one-phase region reduced (shorter composition path) with increased PSf molecular weight, indicating an increase in thermodynamic instability and faster liquid-liquid demixing with molecular weight (Figure 10). A shorter composition path was also observed for solutions containing PEG, which signifies a lower thermodynamic stability than their additive-free counterparts.

**Figure 11:**
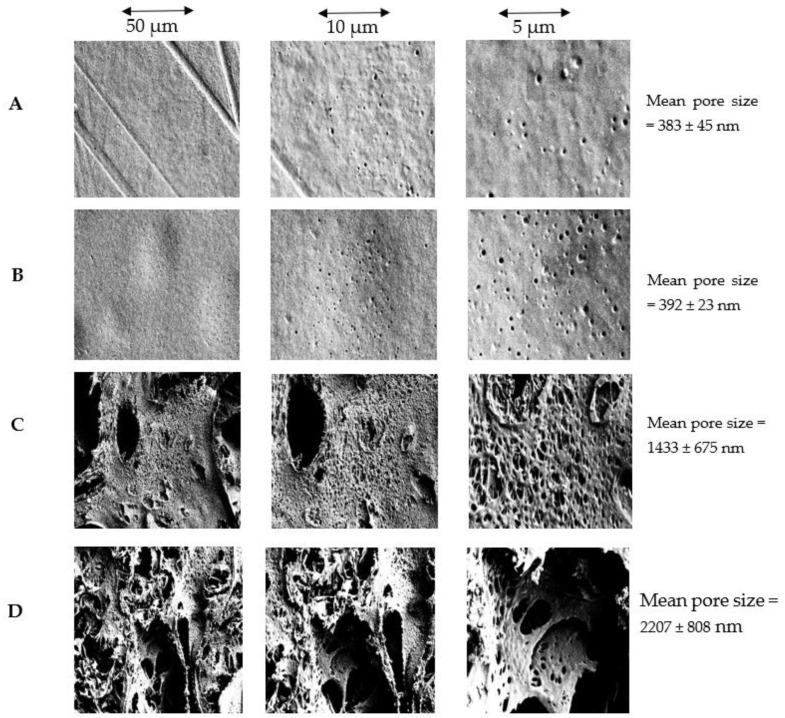
Surface SEM images of (**A**) PSf35 (P1) (**B**) PSf60 (P2) (**C**) PSf35:PEG20 (P7) (**D**) PSf60:PEG20 (P9) at magnifications 1000× (50 µm), 2500× (10 µm), 5000× (5 µm).

The effect of thermodynamic stability on membrane surface pore size and porosity can be observed in the surface SEM images (Figure 11). As seen in Figure 11 and Table 5, the surface pore size increases in the presence of PEG, which agrees with previous studies [31,50]. The surface pores on membranes fabricated with casting solutions with lower thermodynamic stability (P7 and P9) were significantly larger than those on membranes fabricated with more thermodynamically stable solutions (P1 and P2). Additionally, the pore sizes on membranes with PEG were not uniformly distributed compared to additive-free membranes due to the presence of macropores caused by the high inflow of water during phase inversion. P9 was observed to have more surface macropores compared to P7 due to the presence of longer polymer chains (i.e., PSf 60 kDa > PSf 35 kDa) which hinders proper chain arrangement during phase inversion. Higher surface pore sizes and porosity (Figure 11) are responsible for the increased airflow rate across membranes with PEG.

### 3.7. Membrane Mechanical Strength and Filtration Efficiency

**Figure 12:**
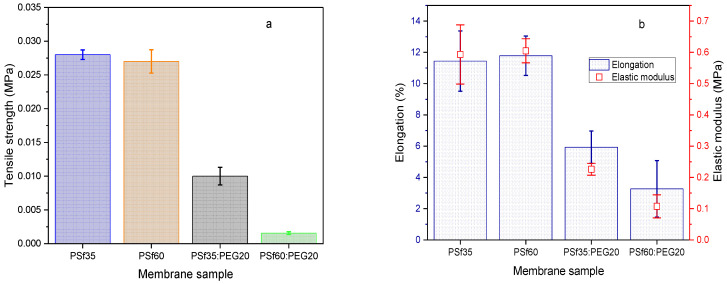
Fabricated membrane mechanical properties. (**a**) Tensile strength; (**b**) Elongation and elastic modulus.

A tensile test was conducted to determine the mechanical strength of the fabricated membranes, which informs their durability and ability to withstand stress. Mechanical strength was expressed in elongation, elastic modulus, and tensile strength. In the presence of an additive, it was observed that the tensile strength decreased with an increase in PSf molecular weight, as seen in Figure 12a. PSf60:PEG20 (P9) showed a significantly lower tensile strength (0.11 MPa) compared to that for PSf35:PEG20 (P7), 0.23 MPa. Similarly, the elastic modulus and elongation decreased with increase in PSf molecular weight in the presence of PEG (Figure 12b). This decrease in mechanical strength with an increase in molecular weight can be attributed to the presence of larger macrovoids in the P9 membrane structure caused by reduced compactness in longer PSf chain (molecular weight = 60 kDa) arrangement in the presence of long-chain PEG molecules. Other studies have reported decreased membrane mechanical strength with larger macrovoids as well [38,55]. On the other hand, there was no statistically significant difference in tensile strength, elastic modulus, and elongation for membranes without PEG (P1 and P2). This could be attributed to the theorized effect of increased PSf molecular weight; i.e., increase in mechanical strength with chain entanglement due to longer polymer chains [56,57] may not be detected for pristine PSf membranes at these conditions (concentration, thickness, and MW difference) using a tensile tester.

**Figure 13:**
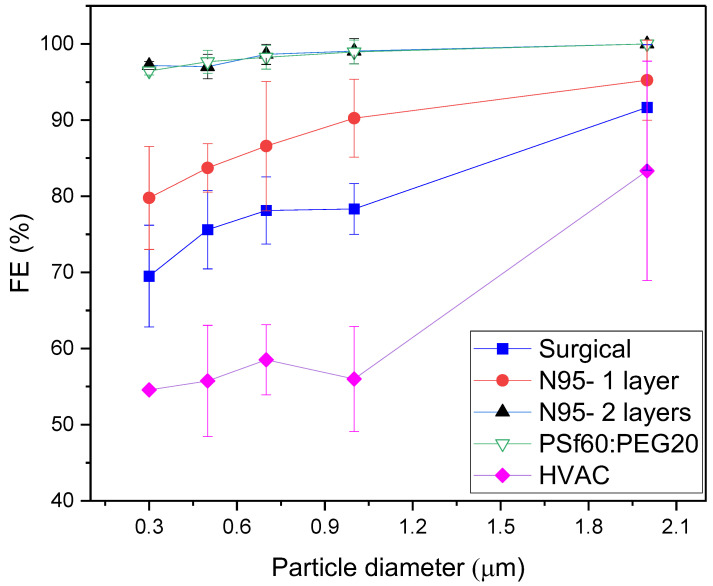
Filtration efficiency comparison between fabricated membrane, P9 (15:15:70 weight percent of PSf60:PEG20:NMP) and commercially available facemasks.

FE values of the fabricated membrane with the maximum airflow and minimum pressure drop, P9, were obtained using aerosolized sodium chloride (NaCl) particles of different sizes and compared with an N95, surgical mask and a minimum efficiency reporting value (MERV) 11 HVAC filter material. FE values of single layer N95 (N95-1), surgical mask (SM-1) and MERV 11 (HVAC) filters, along with double-layer N95 filter (N95-2) were obtained and compared with single-layer fabricated P9 membranes. Among all single layer filters tested, P9 showed the highest FE values as a function of NaCl particle diameter with significantly higher FE values than that of N95-1, SM-1 and HVAC for particle sizes ranging from 0.3–2 µm (Figure 13). On the other hand, there was no significant difference between the FE values of P9 and dual-layer N95 (N95-2). The added N95 layer increases the resistance to aerosol transport, which as expected increases the FE. The high FE values of the P9 membranes could be due to its asymmetric and tortuous pores, which would enhance aerosol capture via sieving, diffusion and impaction [17]. Since the single layer membrane showed greater FE values than single-layer N95 (N95-1) and similar FE values to dual-layer N95 (N95-2), P9 membranes could potentially replace polypropylene non-woven multilayer filters for mask production and MERV 11 HVAC filters.

**Figure 14:**
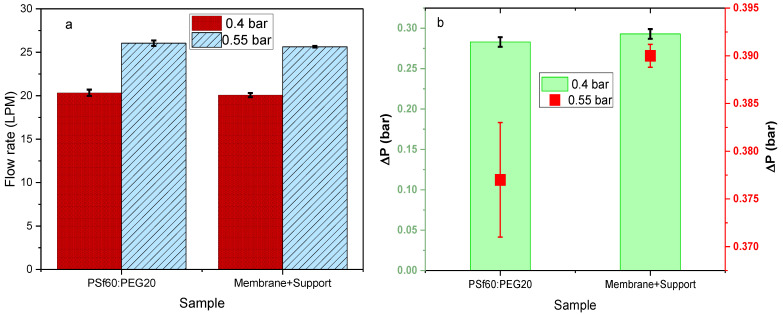
Effect of 3D printed support on (**a**) air flow rate (**b**) pressure drop across PSf/PEG membranes.

P9 membranes with the highest air flowrates and lowest pressure drops at an average of 20.3 LPM at 0.4 bar and 25.8 LPM at 0.55 bar showed the worst mechanical strength when compared to other membrane formulations tested, as shown in Figure 12a,b. Thus, utilization of the P9 membranes for face masks or air filter applications would be impractical since it would likely not withstand any significant stress. To address this, the membranes were attached to a polypropylene (PP) 3D printed mesh support (details in supplementary materials section Figure S4). Airflow, pressure drop, and mechanical strength tests were conducted on the membrane attached to the support (Figure 14a,b). There was an insignificant effect of adhering to the support on flow rate and pressure drop (Figure 14), with average 20.1 LPM at 0.4 bar and 25.6 LPM at 0.55 bar. The tensile strength of the membrane/support also increased by an order of magnitude (0.02 MPa) compared to the membrane alone (0.0016 MPa); hence an increased ability to withstand stress from mishandling and heavy breathing.

## 4. Conclusions

In this work, membranes were successfully fabricated for air filtration by NIPS, using PSf as the primary polymer and PEG as a pore-forming additive. Increased PEG concentration and molecular weight resulted in reduced thermodynamic stability, along with increased porosity and airflow rate; however, the membrane mechanical strength was compromised, making it unsuitable as a filter to be operated under pressure. XPS analyses and contact angle measurements revealed that some PEG remained on the membrane top layer, which likely contributed to the higher hydrophilicity and porosity, and reduced mechanical strength. The fabricated membranes were adhered to 3D printed mesh supports to increase the mechanical strength and resulted in over an order of magnitude increase without significantly compromising the airflow rates and pressure drop. Furthermore, the membrane with the highest flow rate showed similar filtration efficiency values compared to N95 double-layered filters. The single-layer membranes also showed higher FE values than single layer N95 filters, surgical masks, and MERV 11 HVAC filters. The results obtained in this work suggest that porous flat sheet membranes made of PSf in NMP with PEG as pore formers have the potential to be used as face masks/air filters with FE values greater than commercially available masks and HVAC filters, which could also potentially help to protect humans during pandemics.

## Figures and Tables

**Table 1:** Composition of membrane casting solutions.

Membranes	Concentrations (% *w*/*w*)								
	PSF35	PSF60	PEG 1K	PEG 4K	PEG 8K	PEG 10K	PEG 20K	GLY	NMP
P1	15	-	-	-	-	-	-	-	85
P2	-	15	-	-	-	-	-	-	85
P3	15	-	-	-	-	-	-	10	75
P4	15	-	10	-	-	-	-	-	75
P5	15	-	-	10	-	-	-	-	75
P6	15	-	-	-	10	-	-	-	75
P7	15	-	-	-	-	-	15	-	70
P8	15	-	-	-	-	10	-	-	75
P9	-	15	-	-	-	-	15	-	70
P7-5	15	-	-	-	-	-	5	-	80
P7-10	15	-	-	-	-	-	10	-	75

**Table 2:** Surface elemental composition of fabricated PSf membranes.

Membrane	Surface Elemental (mol%)			
	C1s	O1s	S2p	O1s/C1s
P1	86.10 ± 0.95	12.74 ± 0.68	1.16 ± 0.27	0.148
P2	86.88 ± 0.3	12.23 ± 0.29	0.89 ± 0.01	0.141
P7	82.83 ± 1.95	15.55 ± 1.7	1.62 ± 0.28	0.188
P9	82.18 ± 1.5	15.75 ± 1.05	2.07 ± 0.4	0.192

**Table 3:** Elemental compositions of top surface and first etched membrane layer.

Membrane	Oxygen Concentration (%mol)			Carbon Concentration (%mol)		
	Top Surface	1st Etched Surface	% Decrease	Top Surface	1st Etched Surface	% Increase
P1	13.92	5.22	62.5	84.70	92.45	9.2
P2	13.67	5.17	62.2	85.17	92.71	8.9
P7	16.88	4.83	71.4	81.16	92.74	14.3
P9	17.67	4.84	72.6	81.18	93.37	15

**Table 4:** Casting solution viscosity at 0.1 1/s shear rate.

Membrane Sample	Viscosity (Pa.s)
P1	3.8 ± 0.63
P2	4.0 ± 0.77
P4	8.9 ± 1.96
P7	16.1 ± 3.54
P8	11.1 ± 1.43
P9	21.1 ± 4.21

**Table 5:** Surface pore size of fabricated membranes obtained using imageJ.

Membrane	Mean Pore Size (nm)	Max Pore Size (nm)	Min Pore Size (nm)
PSf 35 (P1)	383 ± 45	1151	82
PSf60 (P2)	392 ± 23	878	66
PSf35:PEG20 (P7)	1433 ± 675	18,051	292
PSf60:PEG20 (P9)	2207 ± 808	19,366	412

## Data Availability

The data presented in this study are available on request from the corresponding author.

## Supplementary Materials

The following supporting information can be downloaded at: https://www.mdpi.com/article/10.3390/membranes12070637/s1, Figure S1: Surface images of top left—P7 surface, top right—N95 surface, bottom left—surgical mask surface, bottom right—P9 cross section; Figure S2: FTIR data for PSf35 (P1), PSf35:PEG10 (P8), PSf35:PEG20 (P7); Figure S3: Contact angle images for pristine (a) PSf35 membrane (P1) and (b)PSf35:PEG20 15:15 *w*/*w* (P7); Figure S4. Schematic showing membrane-mesh support attachment; Figure S5. Schematic showing dogbone for mechanical strength test; Figure S6. Surface pore size distribution of PSf35 (P1); Figure S7. Surface pore size distribution of PSf60 (P2); Figure S8. Surface pore size distribution of PSf35:PEG20 (P7); Figure S9. Surface pore size distribution of PSf60:PEG20 (P9); Figure S10. Surface pore size distribution of PSf35:PEG10 (P8); Figure S11. Surface pore size distribution of PSf35:PEG4 (P5); Table S1. Air flow test through membranes fabricated using NaCl as an additive.
