# Supplementary material for: Non-Solvent Induced Phase Separation (NIPS) for Fabricating High Filtration Efficiency (FE) Polymeric Membranes for Face Mask and Air Filtration Applications
Source: Membranes (Basel). 2022 Jun 21;12(7):637. doi: 10.3390/membranes12070637 (PMC9317255; doi:10.3390/membranes12070637)
Supplement: Supplementary file 1 [file membranes-12-00637-s001.zip › membranes-1732316-supplementary.pdf]

membranes

MDPI

# Supplementary Information

## S1.1. Scanning electron microscopy (SEM) images

SEM surface images were taken using an FEI Helios machine (Nanolab 660, Thermo Fisher Scientific, Waltham, MA, USA) as described in Section 2.4. As observed in Figure S1, membranes and commercially available facemasks have different surface structures since the former were made via NIPS while the latter was made using nonwoven polypropylene. This affects the filtration mechanisms and the filtration efficiency. Also, the cross-section of PSf35:PEG20(P7) showed finger link pores with larger voids compared to voids in membranes with lower PEG molecular weights as seen in Figure 7.

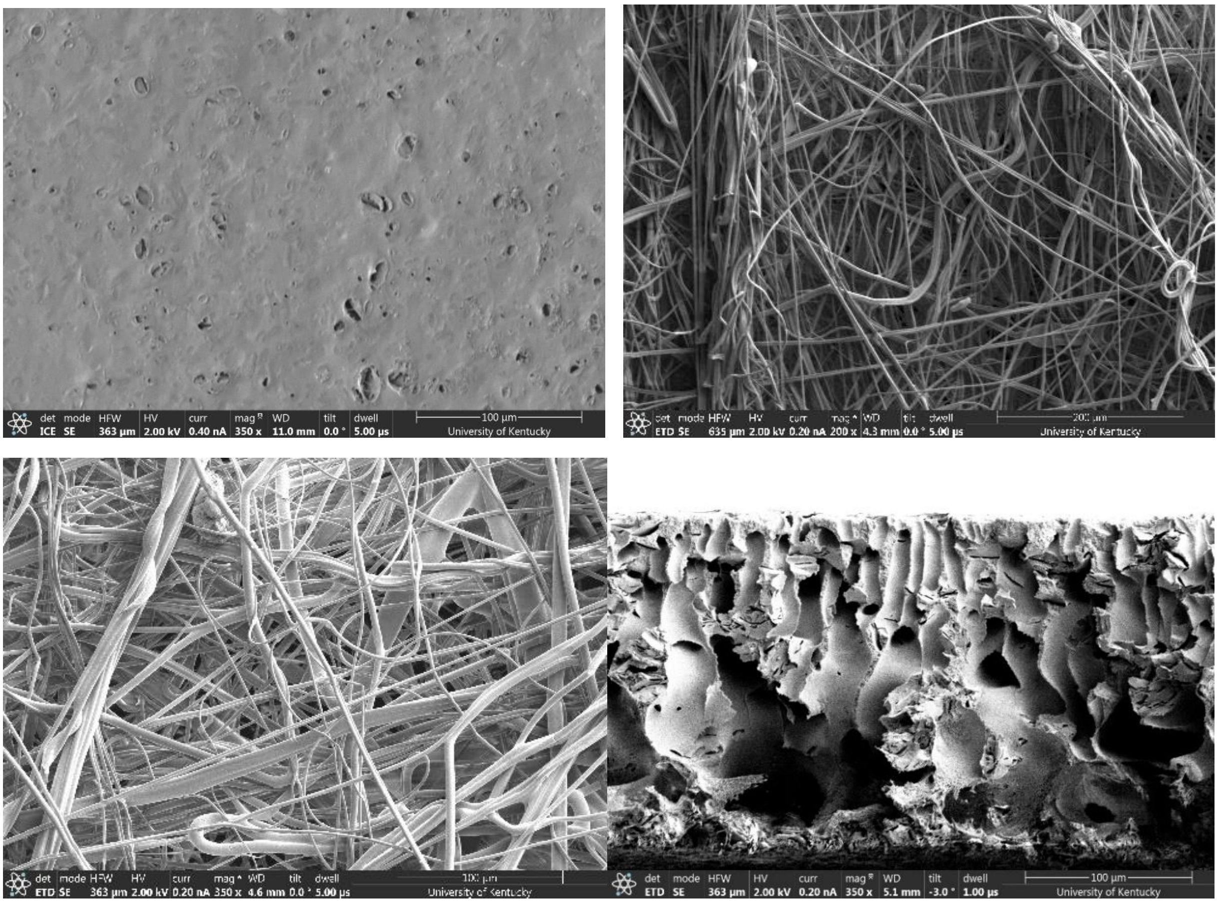


Figure S1: Surface images of top left – P7 surface, top right – N95 surface, bottom left – surgical mask surface, bottom right – P9 cross-section

## S1.2. Fourier Transform Infrared Spectroscopy (FTIR) spectra

FTIR was used to characterize the composition of fabricated membranes using an attenuated total reflection- Fourier transform infrared (ATR-FTIR) (Thermo Nicolet iS50 FTIR Spectrometer, Thermo Scientific, Waltham, Massachusetts, USA), as shown in Figure S2. The IR spectra for PSf35(P1), PSf35:PEG10(P8), and PSf35:PEG20(P7) had similar absorption for all wavenumbers except for 2870 $cm^{-1}$. The strong absorption at 2870 $cm^{-1}$ for P7 and P8 is due to the presence of a CH bond originating from polyethylene glycol, which suggests that membranes made using solutions with PEG resulted in composite membranes.

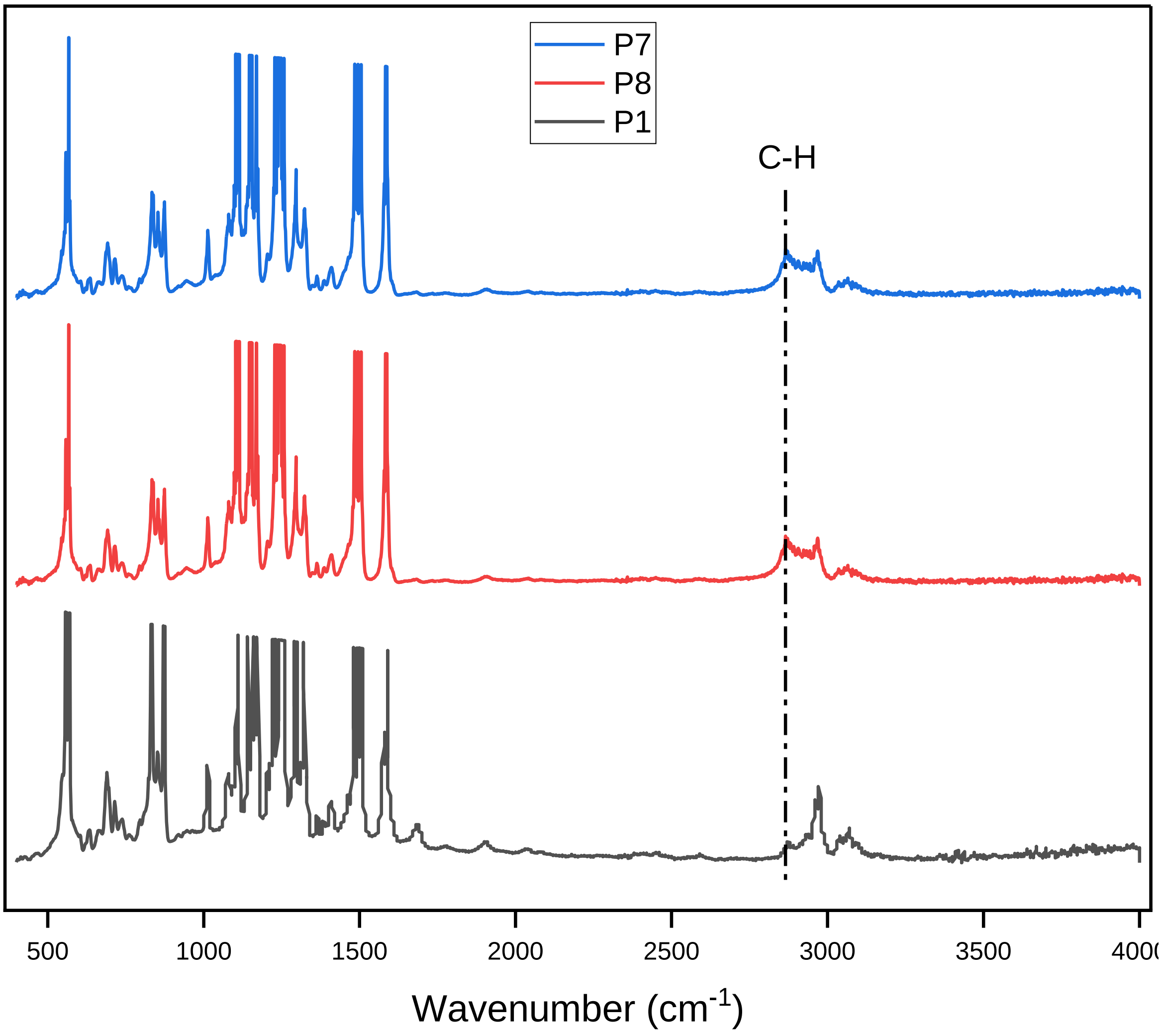


Figure S2: FTIR spectra for PSf35 (P1), PSf35:PEG10 (P8), PSf35:PEG20 (P7).

### S1.3. Contact angle (CA) images

As described in Section 2.3, membrane wettability was determined by using a drop shape analyzer connected to a high-definition camera (Kruss DSA100, Matthews, NC, USA) by estimating the contact angle between a sessile drop and a flat membrane sample. One drop of water of 12 μL was deposited on the membrane surface. The interface between the water drop and the membrane surface was captured by the camera and the contact angle was calculated according to the image, as shown in Figure S3a and b.

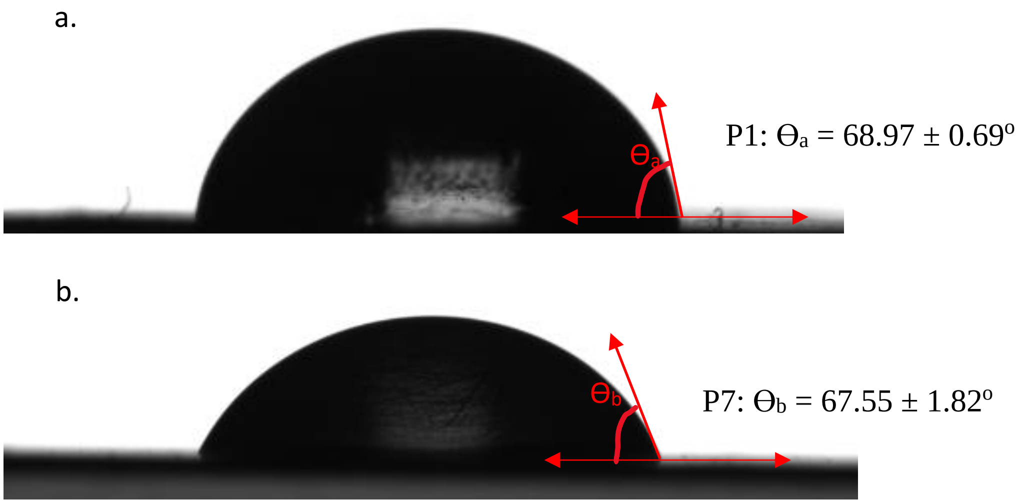


Figure S3: Contact angle images for pristine (a) PSf35 membrane (P1) and (b)PSf35:PEG20 15:15 w/w (P7).

### S1.4. Effect of NaCl as pore former on air flow rate

Sodium chloride was also used as a pore former to observe the effect of a salt on air flow rate, as discussed in Section 2.1. The results, shown in Table S1, were obtained for PSf35:NaCl:NMP (15:10:75)%w/w. No significant airflow was obtained after three trials (Table S1), which suggests that NaCl had no significant effect on pore size at 0.4 bar (6 psi) and 0.55 bar (8 psi).

Table S1: Air flow test through membranes fabricated using NaCl as an additive.

| Trials | 0.4 bar | 0.55 bar |
|---|---|---|
| 1 | 0 LPM | 0.18 LPM |
| 2 | 0 LPM | 0.02 LPM |
| 3 | 0 LPM | 0 LPM |

### S1.5. Polypropylene 3D printed support

The 3D printed support used for this work was printed by the Additive Manufacturing Center at Somerset Community College using the following settings:

- Filament used: FormFutura Centaur Polypropylene
- Retraction – turned on
  - Retraction distance – 5 mm
  - Extra restart distance – 0.00 mm
  - Retraction vertical lift – 0.2 mm
  - Retraction speed – 3600 mm/min
- Layer Height – 0.1- 0.15 mm
- Extrusion multiplier – 1.00
- Extrusion width – 0.40 mm
- Top and bottom layers – 0

- Outline/Perimeter shells – 4
- Infill percentage – 50% - 98%
- Infill type – Grid
- Infill overlap – 25%
- Extruder/Nozzle temperature – 230-240 °C
- Bed temperature – 60 – 85 °C
- Cooling – Turned on – 50% - 100%
- Speed – 50 mm/sec (3000 mm/min)

The membranes were attached to the 3D printed support using an adhesive (Loctite AA3035, R.S. Hughes, Kemper, Cincinnati, OH, USA) compatible with the PSf membrane surface (Figure S4). The adhesive was thinly and uniformly spread along the edges of the support to avoid clogging of the support pores using a polyester tipped applicator and was allowed to dry for 1 hour at 30°C in a convective dryer to ensure a completely dried adhesive which releases insignificant and nontoxic amounts of alcohol and methyl methacrylate before testing for air flow, pressure drop and tensile strength.

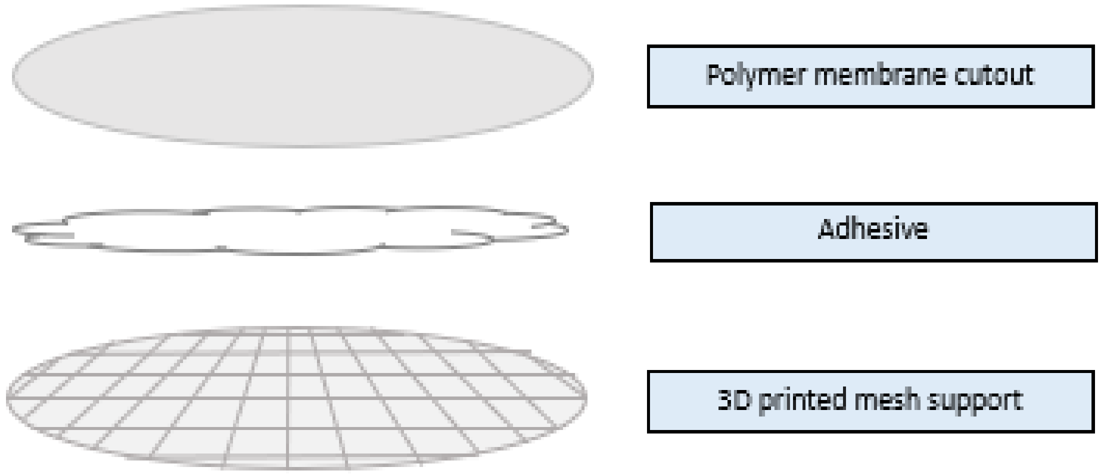


Figure S4. Schematic showing membrane-mesh support attachment

### S1.6. Mechanical strength test

Fabricated membranes were cut into dogbone shapes (Figure S5) for mechanical strength test in a tensile test as described in section 2.3.

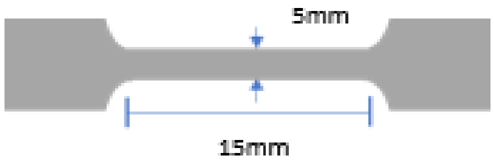


Figure S5. Schematic showing dogbone for mechachanical strength test.

### S1.7. Pore size distribution

The pore size distribution and average pore size measured at magnification 1000X (50 µm) using SEM images and imageJ are represented in Figure S6–S11.

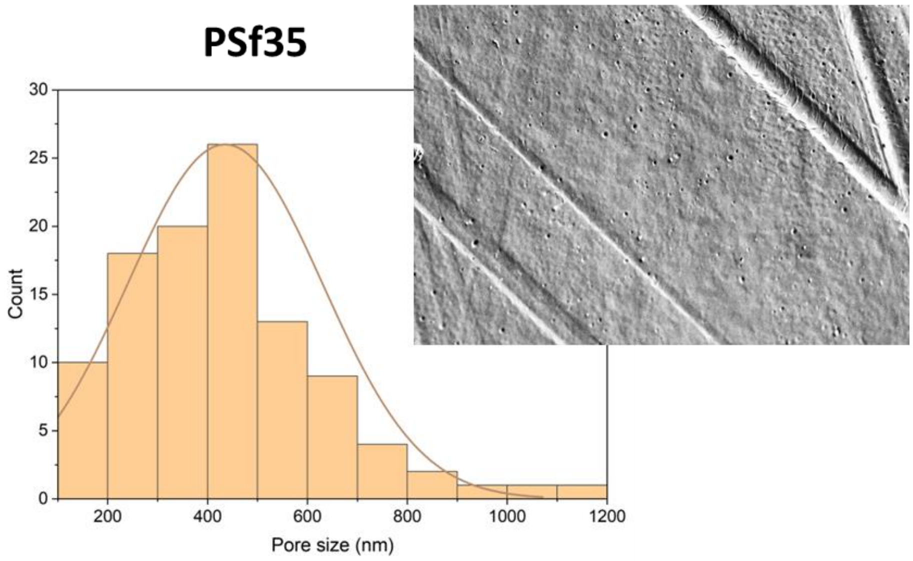


Mean pore size
= 435 ± 195 nm

Figure S6: Surface pore size distribution of PSf35 (P1).

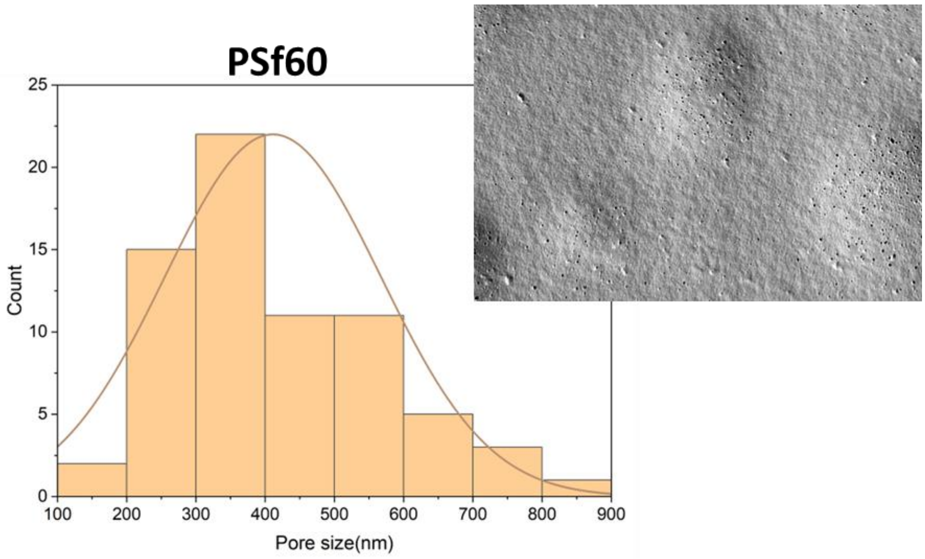


Mean pore size
= 411 ± 155 nm

Figure S7: Surface pore size distribution of PSf60 (P2).

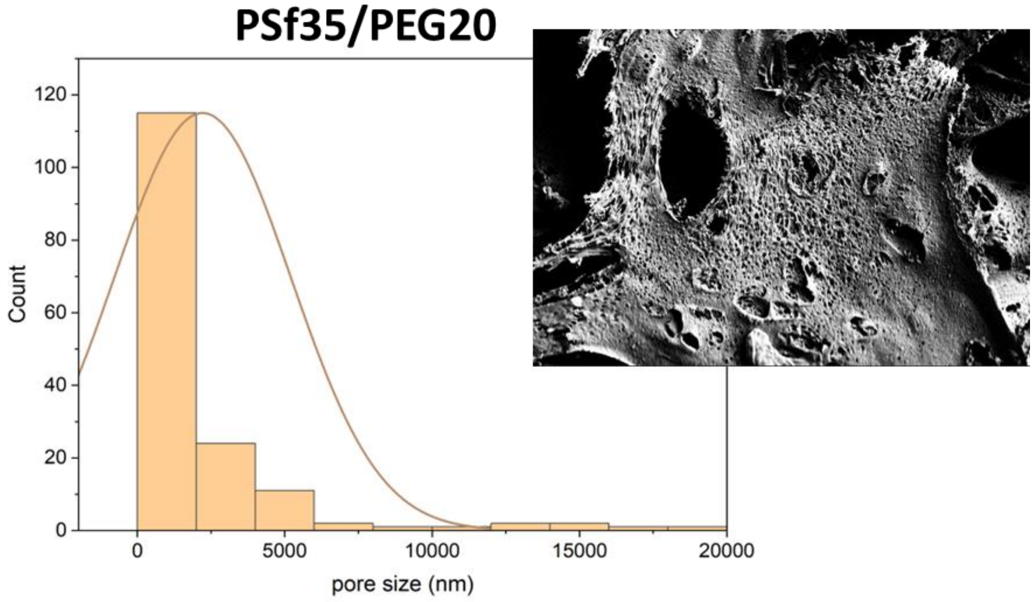


Mean pore size
= 2213 ± 2994

Figure S8: Surface pore size distribution of PSf35/PEG20 (P7).

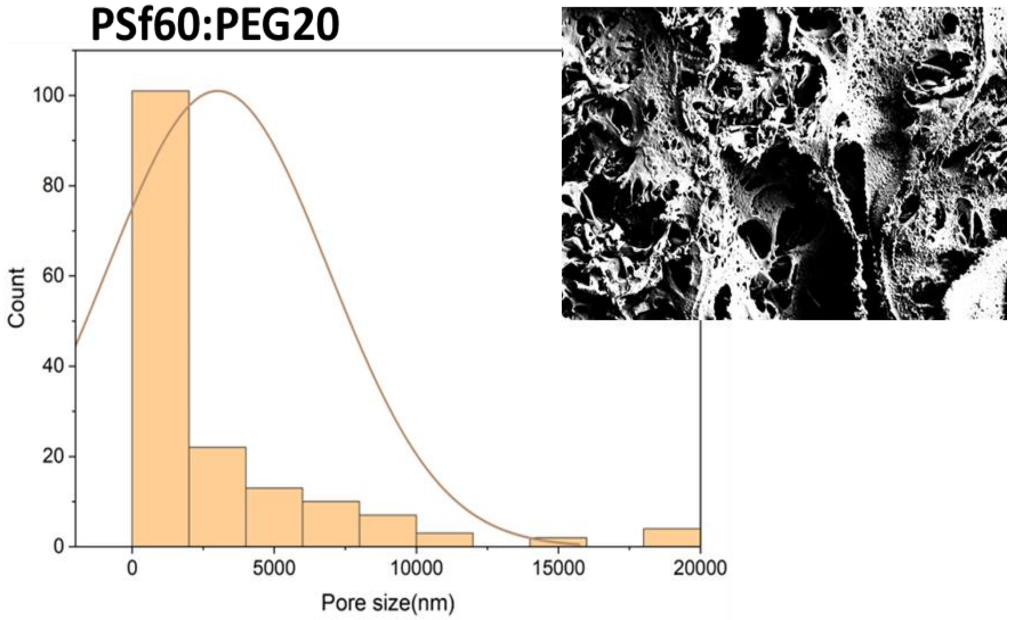


Mean pore size =
3012 ± 3890 nm

Figure S9: Surface pore size distribution of PSf60/PEG20 (P9).

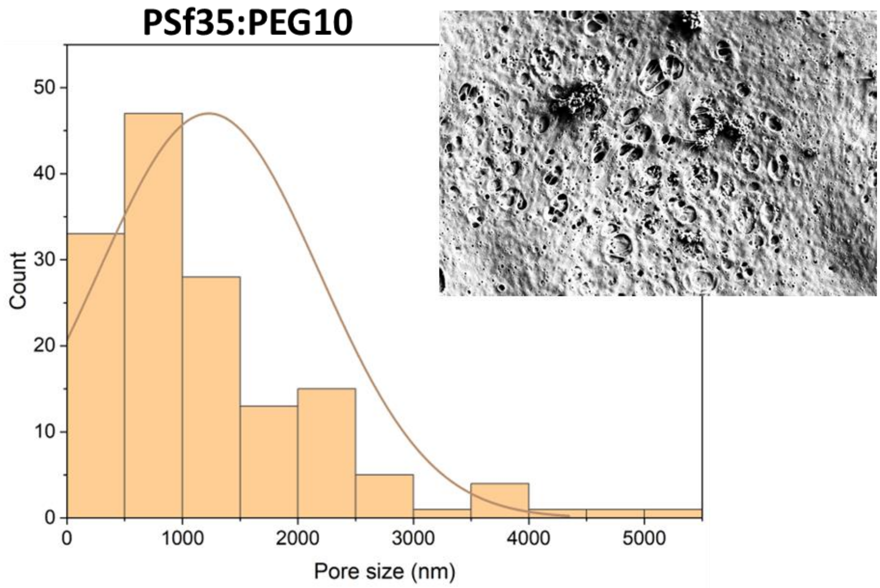


Mean pore size
= 1231 ± 962 nm

Figure S10: Surface pore size distribution of PSf35/PEG10 (P8).

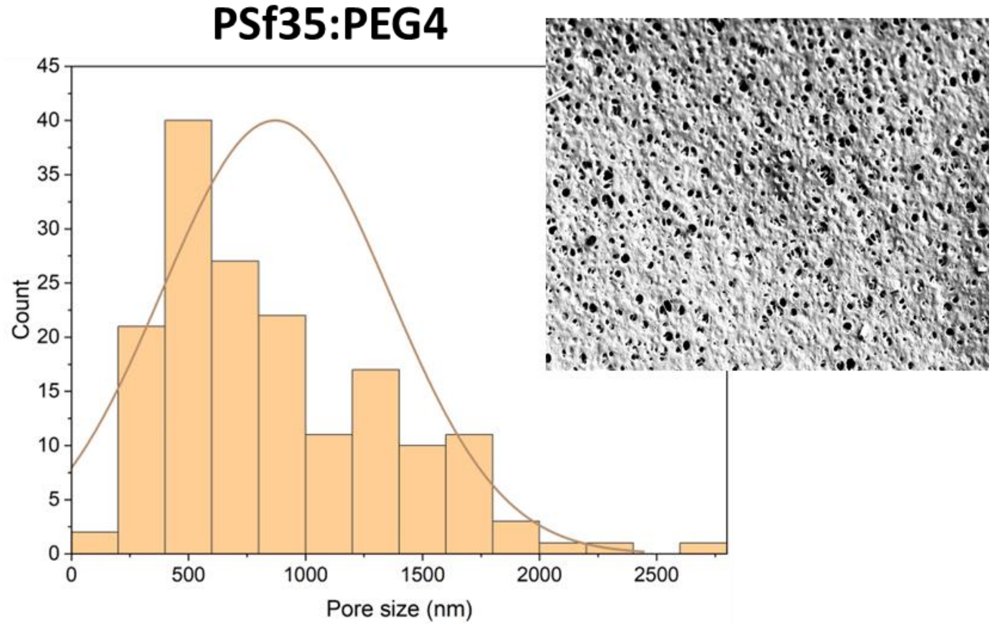


Mean pore size
= 873 ± 486 nm

Figure S11: Surface pore size distribution of PSf35/PEG4 (P5).
